# "The Hospital" Nursing Mirror

**Published:** 1900-11-24

**Authors:** 


### "The Hospital" Nursing Mirror.

Being the Nursing Section of "The Hospital."

[Contributions for this Section of "The Hospital" should be addressed to the Editor, "The Hospital" Nursing Mirror, 28 & 29 Southampton Street, Strand, London, W.C.]

### Notes on News from the Nursing World.

### DUBLIN NURSES AND LORD ROBERTS.

Great interest is being taken at present in Ireland concerning the diamond star to be presented to Lord Roberts on his return by the women of Ireland, in gratified recognition of his valuable services in South Africa. Nurses are having their share in the presentation, and Mrs. Kildare Treacy, of the City of Dublin Nursing Institution, and her staff, last week forwarded to Lady Ashbourne their contributions. Lord Roberts has been specially interested in the institution, and was present in August 1898 at the Viceregal Lodge, Phœnix Park, when the decoration of St. John of Jerusalem was conferred on six nurses in recognition of their bravery displayed in nursing typhus fever cases that year at Inniskea. Snapshot photographs were taken of those on the platform, who included Lord Roberts, Lord Cadogan, the Lord Chancellor and the late William Ireland de Courcy Wheeler, whose memory will be always green amongst the nurses of the City of Dublin Nursing Institution as their benefactor and friend. Nine of the nurses who forwarded their contributions to Mrs. Kildare Treacy have been nursing at Pretoria when Lord Roberts was there, and it was a C.D.N.I. nurse who had charge of Lord Roberts' son until his death.

### PLAGUE IN SOUTH AFRICA.

It is still possible that some of the nurses now in South Africa may be required to remain in order to attend to plague cases. It is satisfactory to learn that the Colonial Secretary at Cape Town, having anticipated an outbreak, has for some time past "retained tents, medicines, and nurses at various centres." The latest reports are also reassuring, and tend to warrant the hope that the disease will not spread beyond the natives at King Williamstown. But if more nurses are wanted there will, we are sure, be plenty of volunteers among those who are on the spot.

### A WELL-DESERVED COMPLIMENT.

The statement of the Secretary of the Local Government Board, endorsed by the Secretary for Scotland, that "Great Britain lies under a heavy obligation to Glasgow for the promptitude and efficiency of the sanitary measures taken by their local authority, their officials, and staff in the presence of a national emergency," includes, of course, a testimony to the manner in which the plague patients were nursed at the Belvedere Hospital. We congratulate the Matron of that admirably managed institution upon the fact that she and her excellent nursing staff are now free from the anxiety which, ever since the first patient suffering from plague was received in the hospital, must have been considerable.

### IMPROVEMENTS AT THE MADRAS GENERAL HOSPITAL.

We learn with pleasure from our correspondent "Pro Bono Publico," whose letter appears in another column, that the way in which the Madras General Hospital has been brought to public notice through "The Hospital Nursing Mirror" has had "the immediate effect of improving the conditions of the nurses in many little ways." This, our correspondent says, gives rise to the hope that they are forerunners of permanent improvements on a large scale, and we trust that the hope will not be disappointed. An inquiry instituted by Government is, in any case, desirable, since it would probably be the best means of ascertaining the precise nature of the changes which are evidently required in order to bring the Madras General Hospital into a line with the best managed institutions in England and her possessions.

### THE GOOLE GUARDIANS AND MR. KENNEDY.

The Goole Board of Guardians are very angry with Mr. H. G. Kennedy, the Local Government Inspector, because he has reported adversely upon the nursing arrangements at Goole Workhouse Infirmary. At a meeting of the Board the other day the chairman traversed Mr. Kennedy's statements, and denied that 32 cases were left during the night to the care of pauper helpers, and affirmed that there were only 48 cases in the Infirmary, and of these only 16 required a nurse. Proceeding, he said: "It was an abominable thing for Mr. Kennedy to say they were neglecting their inmates. They had spent since he had been a member of the Board, between £6,000 and £7,000 on Mr. Kennedy's fads. The only excuse he could make was that Mr. Kennedy was ill when the report was written." Another guardian declared that in no cases were the patients requiring a nurse ever left without one, and in other instances in which Mr. Kennedy said inmates were left to pauper help, the wardsmen and wardswomen were selected with a view to their fitness. Judging from Mr. Kennedy's reports, he is able to take care of himself, and we do not think that the criticism to which he has been subjected will deter the representatives of the Local Government Board from doing their duty.

### VISITING NURSES FOR THE MIDDLE CLASSES.

We have followed with interest the correspondence in our pages on Visiting Nurses for the Middle Classes. It seems to us that some of these who have written letters have not quite grasped what was intended by the correspondent who stated that 3s. 6d. an hour was too much to ask of a working woman of the middle class. It obviously is too much; everyone who knows the usual struggling life of such a woman will acknowledge that. On the other hand, the nurse's point of view has also to be considered, because she, too, is a working woman. But the writer who urged the arrangement of cheaper rates for visiting nurses did not exactly intend that visiting nurses should do the work entirely "off their own bat," to use a slang expression. Her idea was that visiting nurses' associations might be started on the same lines as the district nursing associations now in existence, and in this way she suggested that it might be possible to make a very considerably smaller charge to patients than 3s. 6d. an hour. It must be remembered that 3s. 6d. an hour mounts up by the end of the week to £1 4s. 6d. if the nurse goes merely for an hour a day. This is a large sum to be paid by a woman who is, perhaps, only earning 30s. or £2 a week, or even less, and also has to pay her board and lodging as well.

### MIDDLE-CLASS NURSING IN CORNWALL.

THE Redruth District Nursing Association Committee have again proved that under certain circumstances the nursing of the sick poor and middle-class nursing can be successfully combined. The advantages to the latter, and the benefit of consequent contributions to the funds of the Association, have in no way been allowed to interfere with the efficient nursing of the sick poor, who are attended free of cost, and whose claims are first recognised. In a district like Redruth the sick poor are not always so numerous as to take up all the time of the trained nurse. Payments for services this year form about a seventh part of the total income of the Association—a matter of some little importance—although very secondary to the real good done, and to the genuine interest and good feeling evoked on the part of those attended. Miss Brindley, the painstaking district nurse, having resigned after a residence of nearly six years, will be succeeded by Miss M. Heal, late of the Tavistock Cottage Hospital. The founder of the Association (Mrs. A. Lanyon) has from time of formation acted as treasurer and honarary secretary.

### PRIVATE NURSING IN CANADA.

ENGLISH nurses who go out to Manitoba must be prepared for cold weather and plenty of work. "The nursing," writes a correspondent, "is rough, 'backwoods of Canada,' wooden houses, small, and oh! such dirty people. I only get 10 dollars a week, which is £2. By Christmas I hope to move on to Vancouver; it is rather damp there, but warmer, and one can wear the same clothes one does in England. The best time to come out is in the spring. There are more people of influence in Vancouver, and nursing is better paid—from 3 to 5 guineas a week. Nurses, too, are well treated, but they are expected to work. The doctors are very considerate, and they seem to appreciate fully the fact that, as I overheard one say, 'English nurses can be depended upon to carry out orders faithfully and thoroughly.'"

### READING QUEEN VICTORIA NURSING INSTITUTE.

IT is an unpleasant surprise to find that the Queen Victoria Nursing Institute at Reading does not meet with adequate support from the people in the town. As to its benefits there is no question. At the annual meeting the record of work done showed that during the twelve months 286 patients were attended, and that upwards of 10,000 visits were paid by the nurses; and the report further says, "In every case comfort and helpfulness were afforded, whilst in many cases great additional suffering would have been experienced but for the competent nursing thus rendered." Several individuals have also given substantial assistance, and there is notably one subscriber who, though not a resident of Reading, regularly contributes £50 a year. But the small subscribers, who should be the mainstay of the Institute, are comparatively few in number; and there was a balance to the bad last year of between £30 and £40. Reading, it may be hoped, will take care to remove this reproach before the next annual meeting.

### A NURSING HOME IN A NEW LIGHT.

AN attempt is being made in Glasgow to prevent a house in Claremont Terrace from being used as a nursing home. The objectors, who are seeking an interdict, contend that the premises have practically been converted into a trading place for money-making; that great additional traffic will be entailed by the arrival and departure of patients, nurses, consulting doctors, patients' friends, funeral undertakers, and all manner of tradesmen, which constitute a nuisance; and that unpleasant sights and offensive odours occasion discomfort to the inhabitants adjoining. Finally, the pursuers say that "they are shocked in their feelings and disturbed in their mind by knowing that disease and death constantly prevail in a neighbouring house." The reply of the owner is that not more than twelve patients of the better class will be accommodated, and nine nurses; that there will be no unpleasant sights nor sounds nor odours of drugs; no excessive traffic; and that the house is not intended to be a direct source of profit to him, and at most it may clear its own expenses. He denies that there will be any real annoyance or nuisance, and holds that there is no reason why a well-conducted nursing home should be a nuisance.

### "RETURNED FROM LADYSMITH."

MISS A. F. GRIST, Superintendent Sister of the Army Nursing Service, writing from Cambridge Hospital, Aldershot, asks us to assist her in putting a stop to an imposition. It has been brought to her notice by letters to herself and others at Aldershot that a person calling herself "Green," and saying that she nursed in Ladysmith and belonged to the Army Nursing Service, is calling upon ladies and obtaining money. Miss Grist therefore wishes to say that "there is not, and never has been, a sister of the name of Green in the service." In these circumstances if the person who thus describes herself continues to represent herself as a member of the Army Nursing Service, any of our readers to whom she may happen to appeal will know how to deal with her.

### THE LUTON TRAGEDY.

IT is seldom, we are thankful to say, that nurses give way to intemperance; but the painful case of a young girl at Luton Workhouse Infirmary, who committed suicide by poisoning herself, as the result of taking to drink persistently, is a reminder of the terrible consequences of yielding to temptation. At the inquest it was stated by Dr. Rose that she did her work well, "but the craving for drink was evidently a disease, and it was not safe to leave the patients in her hands." It is unnecessary to dwell on such a sad event, but we cannot help thinking that the authorities at Luton in their anxiety to save the unfortunate young woman from dismissal allowed the patients to run a risk to which they ought not to be exposed. However one may naturally desire to afford a person who gives way to drink opportunities for reformation, no nurse who shows such proclivities should be permitted to remain for a day in a position where a mistake may involve the sacrifice of a life.

### NURSING IN THE ISLE OF MAN.

AT the annual meeting of the Castle Douglas and District Nursing Association attention was called to the fact that a very considerable proportion of the income had been collected in small sums. The Chairman said it was most gratifying that every year they were improving their position, and that this year they were £25 to the good, instead of being on the wrong side as some had anticipated. Another matter for congratulation was that the establishment of the Cottage Hospital, which many people thought would damage the Association, has not had that result.

Both the Association and the Cottage Hospital seem, in fact, to be flourishing.

### A NURSE FOR UXBRIDGE.

The Committee of the Uxbridge and Urban District Nursing Association have secured the services of a competent resident nurse which will, no doubt, be greatly appreciated by the poor of the neighbourhood, particularly in the winter months. A number of subscriptions have been promised, but the Committee are appealing for further financial support in order to enable them to pay their way.

### MIDWIFE OR MATERNITY NURSE!

The members of the Rugby District Nursing Association are divided in opinion as to whether they should secure the services of a maternity nurse, or employ a certificated midwife. Several ladies assert that the representatives of the medical profession in the town are strongly opposed to the appointment of a midwife; and it is stated that five out of six have expressed themselves favourable to that of a maternity nurse. At the annual meeting it was found so hopeless to attempt to come to a conclusion that it was decided to refer the issue to the working committee. We observe that one of the speakers flatly declared "that a maternity nurse would not meet the case." But, as it has been found over and over again in other places, the services of a fully qualified maternity nurse do meet the case.

### THE LADIES' SANITARY ASSOCIATION.

Nurses who work in a district, or think of becoming sanitary inspectors, may be glad to hear of an opportunity offered by Miss Rose Adams, Secretary of the Ladies' Sanitary Association, 22 Berners Street. Unfortunately, the society, in spite of the favourable auspices under which it was started, comes to an end next month owing to lack of adequate support. The entire stock of tracts, books, diagrams, and models is therefore now for sale at half-price, as well as a large supply of cards *re* infectious diseases, bodily life, help at hand, Welsh tracts, and Roth's gymnastic skirts.

### MEMORIAL TO MARY KINGSLEY.

It has been decided to do honour to the good work of Mary Kingsley by a twofold memorial. There is to be established at Liverpool a small hospital for the treatment of tropical disease in connection with the Liverpool School of Tropical Medicine, and there is also to be formed a "Mary Kingsley Society of West Africa," to stimulate research and to collect from all sources information concerning West Africa. A strong financial committee has been formed, but Mrs. Humphry Ward and Mrs. Bishop are as yet the only lady members.

### A NOVEL USE FOR THE DISTRICT NURSE.

The other day, in response to a message left at a District Nursing Home in London, one of the nurses called at a respectable house in a respectable street. There was no one ill, she was told. "But," she said, "did you not send for a nurse?" "Yes," was the reply. "My mother has been ill, but she is better, and quite able to get about; but as she is not strong, we thought it would be nice if you would just come and clean up the house!' The nurse, after explaining that this did not form part of the duties of a district nurse, took her leave.

### "SHE WEARS A CROSS."

An amusing instance was lately given by a staunch Methodist for refusing a subscription to a nursing fund. "No!" he said, "I will not subscribe. The nurse is a Roman Catholic." "Indeed she is not; you are quite mistaken," was the reply. "She *is* a Roman Catholic: she wears a cross," repeated the old man doggedly. Then ensued an explanation that it was the Queen's badge nurse wore suspended by a cord—not a cross; but all to no purpose. "I will not give a penny. She is a Roman Catholic: she wears a cross," were the last words, uttered in determined tones. One cannot convince a man against his will, and evidently to this stubborn mind the nurse's brassard will ever remain a sign and a symbol of the Roman Church!

### "A NEW CURE FOR ERYSIPELAS."

The following amusing little incident happened to a district nurse in the West of England the other day: "I was asked," says our correspondent, "to go and see a man who had a bad leg. The house where he lived was a dirty, disreputable place, and I was warned beforehand that there were large holes at intervals up the staircase through which I might descend into the lower regions if I were not careful. The man's leg was very much swollen and painful, and as I was afraid of 'Plebitis' I advised them to send for the doctor, and promised to call the next day. I went in the morning to find the family assembled in the kitchen, in the centre of which was a man, whom I knew by sight well, a most extraordinary-looking individual, about 3 ft. 6 in. in height: he has no proper legs, but his feet, which are of extra size, with the toes pointing behind, are joined on where the knees should be; this man was 'holding forth' to them. On seeing me one of the women came forward and informed me that they should not need me again, as they had called in this man, 'who was very clever, and had had great experience with legs, and understood all about them.' He had pronounced the trouble to be 'erysipelas,' and was going to cure it with the 'prayer of faith.' Of course I retired vanquished, and left the leg to be cured by a man who had not any himself, but 'had had great experience with them.'"

### THE MATRONSHIP OF MELBOURNE HOSPITAL.

The treasurer of Melbourne Hospital, who is now in London, informs us that he has selected from among the number of applications received three, and has forwarded the testimonials of the candidates, with his report thereon, to the Committee of Management. Mr. Godfrey himself expects to be in Melbourne before the end of the year to confer with the Committee upon the appointment.

### OUR CLOTHING DISTRIBUTION.

We have to acknowledge the receipt of a parcel from Nurse A. Hillman, Charmouth, Dorsetshire, containing some very acceptable stockings, warm gloves, and other useful articles. The announcement of Nurse Hillman's welcome contribution will, we hope, act as an incentive to others of our readers to forward any gifts they intend to send as soon as possible, and in any case not later than Monday, December 17th.

### SHORT ITEMS.

The s.s. *Orcana* has again arrived at Southampton with sick and wounded. The nursing staff consisted of Acting-Superintendent Sister Mark, A.N.S., and four reserve nurses — sisters Rees, Carter, Tyrell and Atkins.—The North Bierley Board of Guardians have appointed a committee to consider an application from the proposed Shipley District Nursing Association for financial support on the ground that the scheme will be the means of saving the rates.

### Lectures on Nursing for Probationers.

By E. MacDowel Cosgrave, M.D., Lecturer to the Dublin Metropolitan Technical School for Nurses.

No. XIX.—ON FOODS.—(*continued.*)

The last lecture dealt with four of the classes of food, viz.:—Proteids, carbohydrates, fats, and salts. The fifth and remaining class is—

V. Water.—This is a very important food, as three-quarters of the body-weight is made up of water, and only one quarter of all other substances. Water is necessary for digestion, as it is only when food is dissolved that it can be absorbed; it is necessary for circulation, which consists of water currents bearing oxygen and dissolved food-stuffs throughout the body; it is necessary for excretion as urea and all other substances have to be got rid of in a state of solution; the regulation of temperature by evaporation from the skin necessarily causes a loss of water which has to be replaced.

A great deal of water is contained in what is often spoken of as "dry food;" thus water forms of uncooked bacon a quarter of its weight; of mutton half its weight; of chicken three-quarters its weight; and of tripe four-fifths its weight. In vegetables the proportion may be still larger, as water forms nearly ninety-three parts in the hundred of the turnip, and ninety-six parts of lettuce, leaving in the latter case only four parts in the hundred for all the other constituents.

All these classes of food are needed:—

Too little proteids causes muscle waste and loss of strength.

Too little fat causes lessened heat and force, and leads to loss of flesh.

Too little carbo-hydrates leads to gradual starvation, which no amount of the other classes of food will prevent.

Too little salts leads to rickets, scurvy, anæmia, &c.

If fresh vegetables are not used symptoms of scurvy will appear; the earliest are a pale and flabby appearance, with sponginess of the gums, and bruise marks following the slightest injuries.

If such symptoms appear, green vegetables—such as cabbage, spinage, watercress and lettuce—potatoes (especially when baked), and oranges and lemons should be taken.

As a matter of fact ordinary table salt—chloride of sodium—is the only salt taken separately, as food contains enough of the other substances. In illness, however, other salts are often administered; thus in rickets, the bones not developing well, salts of lime are given, and in anæmia, there being a scarcity of red-blood cells, iron is given.

If too much meat and too little vegetables are taken boils and skin eruptions may appear.

Too little water causes delayed and deficient excretion, impurities accumulate in the blood, the kidneys are irritated by having to remove waste matters insufficiently diluted, and the urine is loaded.

On the other hand, too much food leads to indigestion, loaded bowels, and deposits of fat, and throws an extra strain on the excreting organs, especially the kidneys. Too much carbo-hydrates causes the tissues to become fat and flabby, the flesh not being firm. Too much proteids throws a strain on the kidneys and heart, and leads to gout.

Meat should not be taken too often, probably twice a day is sufficient. Dinner and breakfast or supper—preferably breakfast—are the best times.

Variety is very important in diet. Food may contain all the substances needed for nutrition, yet, after a time, the system may tire of it and be unable to use it. This is especially true of concentrated foods.

Fortunately people seldom tire of bread and butter, meat, potatoes and tea; and the same may be said of the breakfast boiled egg or fried bacon.

A mixed diet is best as there is no article of diet—except milk—which has the different elements in proper proportion, and although milk will do during the enforced rest of illness, a more solid diet is necessary for those going about and doing work.

Each person requires daily about 300 grains of nitrogen and 4,000 grains of carbon. A pound of lean meat contains the right quantity of nitrogen, but four pounds would have to be eaten to get enough carbon; so four pounds of potatoes contain the right amount of carbon, but twenty pounds would have to be eaten to get the required nitrogen.

By using a mixed diet of meat and potatoes, about three-quarters of a pound of meat and three pounds of potatoes give the required amounts.

Experience has led to the use of all these classes of foods at the chief meals. Thus a breakfast of tea, bread and butter and fried bacon, or a boiled egg, has all classes represented, as has also a dinner of water, meat (fat and lean) and potatoes.

The recognition of this necessity is also shown by the way in which certain articles of food are commonly combined; thus chicken, the flesh of which is nearly free from fat, is usually taken with bacon, bread sauce adding a carbohydrate. Bacon also goes with rabbit, and with cabbage and potatoes; indeed the large proportion of fat in bacon makes it a useful addition to many other foods. Again, boiled dumplings and Yorkshire pudding are frequently served with meat. Irish stew is another good example of a dish which includes all classes of food. Curried chicken and curried lentils represent the combination of carbohydrates with animal and vegetable proteids.

The most common defects in diet are:—

1. Deficient nitrogenous foods (proteids). This results partly from the dearness of meat, which is the usual source of our nitrogen, and from not using the cheaper vegetable sources. The ease with which bread and tea can be got ready is another cause.

2. Want of fresh vegetables. Many people think they cannot digest vegetables and so avoid them; this is a frequent cause of ill-health.

3. Want of salts. This occurs chiefly in those who do not take fresh meat and vegetables. White bread, for example, is wanting in phosphites, but these can be obtained from eggs, milk, meat, &c. For this condition fresh meat, fresh vegetables, raw or stewed fruits, rhubarb, oranges, lemons, &c., should be taken. A free use of vegetable soups would greatly improve our national dietary.

4. Want of fat. This is most frequent in children and in delicate adults. Bacon, butter, cream, and suet puddings can often be taken when fat in other forms is rejected.

A few examples of food values may be given:—

Eggs are equal in food value to the same weight of butcher's meat. The yolk is the richest part as it contains more of the fat; it gives nearly six times as much force to the body as does an equal weight of the white.

Rice, which is the main food of one-third of the human race, contains only one part of flesh-formers to ten of heat-givers, and so has too little nitrogen to be in itself a perfect food. Taken with milk, or with eggs, &c., as in curry, this deficiency is made up. It is best steamed till tender, as boiling removes much of its nitrogen and mineral matters, neither of which it can afford to lose.

Oatmeal has one part of flesh-formers to about five of heat-givers, but is wanting in fat, so is best taken with milk.

Many experiments have been made to determine the time required for the digestion of food; the following table illustrates the range of time:—

| | Hours. |
|---|---|
| Tripe ... ... ... ... | 1 |
| Chicken ... ... ... | 2¼ |
| Non-oily fish ... ... ... | 3 |
| Roast beef ... ... ... | 3 |
| Roast mutton ... ... ... | 3¼ |
| Roast veal ... ... ... | 4 |
| Roast pork ... ... ... | 5 |

### Nursing on the "Nubia" Hospital Ship.

A CHAT WITH THE NURSING SUPERINTENDENT.

By a Correspondent.

As the *Nubia* hospital ship had just arrived for the third time at Southampton, I took the opportunity of interviewing the Acting Superintendent, Sister Steel, of the Army Nursing Service. The *Nubia* is one of the finest now being utilised by the War Office for hospital purposes: her twin sister is the *Simla*, and both were chartered and fitted up about this time last year.

"There is every convenience for nursing," said Miss Steel, "the Hospital Commissioners came on board just before we left Durban, and they pronounced her perfect—too luxurious, if anything. In fact, they were quite charmed. She certainly looked very nice indeed; we had plants and flowers then in the wards, which are, of course, impossible when at sea; the ship had been used as a base hospital between her trips."

"How are the wards arranged?" I asked.

"The great feature about them is that they run one out of the other, so that a great deal of walking is saved."

"Is there an operating theatre?"

"Yes, and throughout the ship there is every modern appliance—electric light, fans—particularly valuable in the tropics—and electric kettle, steriliser, &c.; but operations are never performed at sea unless absolutely necessary. One man who had a bullet in his spine, and another in his leg, was very anxious to have them removed, but the doctors refused to undertake it at sea, as the patient suffered very little inconvenience, and it was better to wait for more favourable conditions."

"Were there many serious cases of wounds on board?"

"Not very many latterly, but we have had some frightful cases. One officer came on board this time perfectly helpless, dragging himself along on crutches—no power in the legs at all, but with the voyage and massage he recovered sufficiently to walk on shore when we disembarked."

"Did you give massage much?"

"Yes, a good deal; many of the patients suffered especially in the legs, after enteric, and it did them a great deal of good. I think it will be found that massage is beneficial in many cases where it has not heretofore been used."

"How many stretcher cases were landed?"

"Only ten. The lifts are a very great help, as patients can be brought up from the hospital without any risk of injury."

### Cases of Insanity.

"We have two padded rooms on board," Miss Steel went on, "for one never knows what might happen during a voyage."

"Have there been many cases of insanity?"

"I believe several. We had one on the *Trojan*; a very nice corporal went religiously mad through overstrain and want of sleep. He was left at Wynberg Hospital, as insane cases are not supposed to be sent home on the hospital ships."

"Had you a smooth passage?"

"After leaving Cape Town; but we had a terrible time between Durban and there; everyone was sea-sick. But the Bay of Biscay was like a millpond."

"Who composed the nursing staff?"

"Myself and six Army Reserve sisters. As many reservists as possible are being sent now, and I believe there are still over 600 in South Africa. Our thirty orderlies were not R.A.M.C. men, but St. John Ambulance men and members of the Maritzburg Ambulance Corps. There was a staff-sergeant in charge, one corporal, and two privates to do office work, pack-store, &c."

### Uniform on Board.

In reply to an enquiry regarding the wearing of uniform on board the hospital ships, Miss Steel said decidedly, "I am most particular about it myself, and never wear private clothes on the return voyage except when going on shore with leave of absence. Going out, of course, we have no work unless any of the ship's people are ill, as happened last time, when an orderly developed enteric on board, but even then, I always go to dinner in indoor uniform. On the *Nubia* we have convenience for washing (except starched things); and it is very troublesome to have much luggage in your cabin. I only wear blouses and skirts for convenience. Our P.M.O. is very strict about it, too."

"Have the sisters good and convenient cabins?"

"Yes, they are nice and large, and we have our own stewardess."

"How long have you been Acting Superintendent?"

"Since last May on the *Nubia*; I was nursing on the *Trojan* before, and I was at Cork before that, where I went straight from Netley."

"Were you, like so many, anxious to get to the front?"

"I should have been glad to go; but I was wanted on board, and I have been there all the time, except for short leave, when I went up the country from Durban."

"You are returning to South Africa, are you not?"

"Yes, in a few days."

The life of an Army Nurse is not, Miss Steel says, one of brilliant prospects, for there are only a limited number of openings for promotion, but, like the Post Office Savings Bank, it is a "safe investment;" and there is a pension of £30 a year after the age of 60. It is her opinion that many more nurses will be appointed in the military hospitals, and that the duties of the orderlies will be more strictly confined to the rough work of cleaning the wards, fetching and carrying, &c. Probably the first offers will be made to the Army Nursing Reserve Sisters who have been through the South African campaign.

"The War Office will have to reform many things," she said, "and this war has proved that there are not nearly enough nursing sisters in the service."

### An Object Lesson from Croydon.

By a Matron of Experience.

The action of the Croydon Board of Guardians in reducing the superintendent of nursing to the inferior position of upper nurse (without authority) and general housekeeper, cannot fail to give rise to much speculation on the part of outsiders, and to those of us who know the undercurrents of hospital life to not a few grave surmisings.

Conscientious matrons, sisters, and nurses know, unfortunately but too well that there is a decided tendency towards degeneration in the nursing profession at the present time.

Nurses have increased in numerical strength and in technical knowledge, but there has been wanting an equivalent increase in conscientiousness and the other attributes of character essential to the nurse who can be trusted, and to whom any of us would willingly entrust the care of our loved ones in their hours of danger or extremity.

### The Untrustworthy Nurse.

There is a type of woman very much "en evidence" in the nursing world, and daily becoming more so, who had much better be out of it.

She may be either well born or of lowly parentage, but she is best described by the term "untrustworthy." This young person is usually sharp and very much to the fore when the doctors are concerned—quick to hand the surgeon the instrument or sponge that he requires, ready when asked by the physician to state facts about her medical cases without the slightest hesitation. She apparently seldom forgets, and is never uncertain about anything.

The long-suffering matron, sister, or nurse under whom this paragon works (who out of kindness does not always show her up at the time) knows, however, that her information is frequently utterly incorrect, and that she neglects both her duty and her patients. When the doctor is not present she has to be reminded of everything. Poultices and fomentations are put on either lukewarm or so hot as to endanger the scalding of the patients, feeds are forgotten, temperatures are incorrectly charted, medicines are given in wrong quantities, and the directions misread. Patients are kept waiting for what they require, and their comfort or welfare is a very secondary matter indeed to the nurse's own convenience. To "get through" her period of training with as little work and trouble to herself as possible to the freer and easier life of a private or district nurse or some equally desirable appointment, in which it is to be feared her patients can be more or less neglected with impunity, is the great ambition of such a woman.

A Question of Vital Importance.

At last a nurse of this kind scrapes through her examinations and comes to the end of her three years' period of bondage, and now she expects to receive the certificate which will guarantee her as a properly qualified nurse, fit to hold responsible posts. She has never done anything sufficiently morally wrong perhaps to warrant her summary dismissal from the hospital, and yet she is utterly unfit to have the care of the sick and the issues of life and death in her hands. The question therefore stands thus: Is the public to have these three-years-trained frauds foisted upon them, or by withholding certificates are matrons to have the power of putting a stop to a crying evil of which they alone can be the proper judges?

The Essential Qualifications.

Everyone will admit that nursing is essentially a woman's occupation, a woman's art. Though a doctor here and there specially circumstanced may be able to teach nursing, he is "the exception that proves the rule." The art of nursing was a woman's invention, and doctors were unable to teach it at all till Miss Nightingale and a few other women of the same stamp showed them what it was and might become. To carry out accurately the doctor's orders is only a part of nursing—a most essential part, of course—but there is also the woman's side which a man cannot teach, and knows little about. There is the gentle touch, the womanly tact, the sensitive feeling for your patient, the perfect truthfulness which is brave enough to own a mistake, the absolute conscientiousness and entire devotion to the work without which no one can ever be a nurse in the highest sense of the term. Probationers may be trained for twenty years, but if they do not possess these qualifications they will never be either kind or reliable nurses.

As a matron and a woman I would rather have a probationer of three months' standing to nurse me, had the probationer these essential qualifications, combined with brains and common sense, than a nurse of six years' experience, who had passed every conceivable examination and had learnt everything it was possible to learn about every disease under the sun, who did not possess them.

With the former my life would be in comparative safety, with the latter in hourly danger.

A College for Nurses.

It is much to be desired that ere long all such unseemly friction as that displayed at Croydon Infirmary may be brought to an end by the establishment of some central college which in the nursing profession would take the same place as do the College of Surgeons and College of Physicians in the medical

Here nurses would receive their diplomas after successful examination, such nurses only being eligible for examination as hold certificates of good character and conscientious work from the matron of their hospital or an equivalent testimonial. The examiners being physicians and surgeons from the leading London hospitals and matrons of ability and common sense, there would be little opportunity for favouritism, and each nurse would stand upon her own merits. It seems to me that were such a college of nursing to be established, it would not only have the advantage of putting an end to squabbles, but would also give a recognised standard of nursing at present impossible owing to the variety of methods adopted by the different training schools, the best of which would in every instance be then selected.

### "The Hospital" Convalescent Fund.

It is some time since our readers have heard any particulars in these columns of the work that is being done by the Hospital Convalescent Fund, and we therefore feel sure they will like to hear now how steadily though unobtrusively it continues to help those nurses who are unfortunately in such a position as to require assistance. Many an overworked nurse or one recovering from illness, and without the means to procure a rest, has benefited by the timely aid of "The 'Hospital' Convalescent Fund." A nurse wholly dependent upon her earnings, and not attached to any institution, finds the time of sickness press very heavily upon her resources; and even more trying is the period of enforced idleness due to exhaustion following on overwork, to combat which it is necessary that the sufferer shall have complete rest and freedom from care. This is most necessary to enable her to again earn her bread with credit to herself and her profession, and almost an impossibility when the nurse is run down in health. We have just sent to the seaside a nurse who has been unable to follow her profession since January last, having been a constant sufferer from nervous exhaustion due to overwork and a weak heart. She had been for some time at a home on the East Coast and derived great benefit; but the doctors recommended a further period of rest and sea air in order to complete the cure. This the nurse was unable to afford, as her means had been much depleted owing to the expenses of her illness, and the help of the Convalescent Fund has, therefore, been very much appreciated. She writes to us: "I feel so very grateful to you for sending me here. Words seem so cold and empty; but I must get quite well and strong, and try to be a credit to you." In another letter she says: "I cannot find words to thank you as I ought, but I do indeed thank you very heartily, and I hope when times are better that I shall not forget to help the Fund, for you will never know how dreadful it is to be stranded with no money." Another case which we were able to assist a short time ago was that of a district nurse who contracted diphtheria owing to the insanitary state of the town where she was working. She went to the seaside to recover her strength, which, however, she found a very slow process, and at last her funds were almost exhausted. She could not gain admission to any of the free convalescent homes owing to the infectious nature of her illness, and she therefore applied to us. We were able to send her to a convalescent home for infectious diseases, where she was very comfortable, and her stay there proved of great benefit. She wrote us: "I shall always feel deeply grateful to you for your kindness in sending me here. I do not know what I should have done without this rest."

The nurses whom we have helped do not forget the Fund when they are back at work, and it is very gratifying to receive from time to time unsolicited contributions from them. There must, however, be very many others who could afford a small subscription or donation to "The 'Hospital' Convalescent Fund." Nurses have justly the reputation of being generous, and such a reputation brings many calls which a nurse is not always able to comply with. But there is another way in which she can help her fellow-worker in the time of ill-health and want—namely, by collecting from friends and patients, who would in most cases be only too glad to give a donation to help those to whom they look for comfort and help when sickness attacks themselves. We hope, therefore, that nurses all over the country and abroad, and all those who are interested in nurses, will take an early opportunity of sending a donation to the Honorary Secretary, "The 'Hospital' Convalescent Fund," 28 and 29 Southampton Street, Strand, London, W.C., by whom they will be gratefully received and acknowledged in these columns.

### The Nurse in Hot Climates.

By EDWARD HENDERSON, M.D., F.R.C.S. Edin., late Surgeon, General Hospital, Shanghai.

(Continued from page 94.)

Various details which have special reference to the nurse's duties in connection with fever cases in hot climates have already been sufficiently discussed in what has been said regarding the taking of temperatures, the use of punkahs and mosquito-nets, ice and the cold bath, and I do not propose to add to them here. Prophylaxis against fevers generally will be considered later.

Convalescence after fever of any severity is a slow process among Europeans in the tropics. The muscular and nervous systems regain tone slowly, gastric catarrh lingers, the lost appetite is restored with difficulty, and the skin acts excessively, especially at night. The treatment of these cases taxes the resources of the doctor and the patience and perseverance of the nurse. In the end all efforts may fail and a complete change of climate be found the only remedy. Fresh fruit is a welcome and valuable addition to the diet of convalescents: in giving this to her patients the nurse should remember the precautions advised in her own case in what was written on the subject of her voyage and life on board ship.

*Surgical Cases.*—The duties of a nurse who is in charge of surgical cases abroad do not differ in any important particular from those of a nurse who is similarly employed at home. In very hot weather, when the patient is perspiring profusely, antiseptic dressings may need to be more frequently changed; but this is a matter which only indirectly concerns the nurse. Any appearance of discharge on the surface of a dressing, as from a recently-opened liver abscess, must have immediate attention. When detected by the nurse she should, unless otherwise instructed, at once apply additional protection in the shape of a pad of gauze wrung out of carbolic lotion, laid on the outside of the dressing, and retained in position until the surgeon or his assistant is able to see the case. Inflammatory affections of the liver ending in suppuration are of frequent occurrence in hot climates in connection with the bowel lesions caused by dysentery and the more serious forms of diarrhœa. When an abscess is deeply placed in the liver substance pain is rarely severe, and may be altogether absent; when, on the other hand, the abscess is superficially placed or is approaching the surface, pain becomes a leading symptom, and is usually acute and stitch-like in character. Should a hot fomentation be ordered in a case of the kind, the nurse must be careful in applying it to avoid any injury to the skin. The opening of a liver abscess on the surface of the body is an operation which must be done under strict antiseptic precautions, and a suppurating surface, resulting from a superficial burn or an injudicious application of iodine, might delay or endanger the necessary surgical interference.

If the nurse is at any time put in charge of the instruments she may be warned that in hot climates, when, as often happens, there is much moisture in the air, steel instruments are sure to suffer if not carefully looked after, and a single day's neglect may seriously injure needles and the edges of knives and scissors. At such times it is better, after washing and carefully drying the instrument, to smear it over with paraffin ointment—an ointment in which soft and hard paraffin are combined in proportions easily altered to suit the climate. Vaseline (soft paraffin) has too low a melting point, and in really hot weather is apt to run together and leave spaces exposed on the surface of the instrument. Paraffin ointment keeps well under all circumstances and needs no antiseptic addition; boracic acid in an ointment injures steel if left long in contact with it. If the smearing is done carefully with a suitable ointment, the instrument will go through a whole wet season without sustaining any damage; and this is an important matter in the case of instruments which are only used on rare occasions, and as regards knives in situations where the services of a cutler cannot be obtained. Boiling in water to which a little carbonate of soda is added effectually removes the paraffin. The finer instruments, the cataract knives and scissors, used in eye operations, will need more careful treatment, but these the surgeon is scarcely likely to trust in any other hands than his own.

*Obstetrics.*—Success in the management of obstetrical cases, so far as the nurse is concerned, depends on her ability to maintain the greatest degree of surgical cleanliness which the nature of these cases permits, the difference between a hot and a temperate climate in no way materially affecting the details of the work to be done. In view, however, of the fact that a high atmospheric temperature favours early decomposition, it is advisable that the nurse practising in the East should give a little extra attention to the washing of the patient, and the changing of the pads used to absorb discharges.

In hot weather the labour may with advantage be conducted under a punkah pulled from the outside, usually from a veranda on the ground-floor. When possible an entirely fresh bed should be provided for the patient when labour is completed: this arrangement has many advantages, and the patient, who has probably been perspiring profusely for several hours, will welcome her complete and easily effected removal from wet and soiled surroundings. In midwifery cases the less use there is made of bed-pans the better; in all natural cases the bladder and bowel, if ordinary care be taken, can quite safely be emptied in the sitting position on a suitable receptacle placed at the bedside.

In ordinary cases when the necessary care has been bestowed by the physician in the use of antiseptics during labour, the cleansing of the external parts should be sufficient; and a solution of carbolic acid, the 5 per cent. solution diluted with an equal quantity of warm water, is perhaps as good a lotion as any to employ for the purpose. Absorbent cotton impregnated with salicylic acid is one of the best materials to use for the pads applied to the vulva to absorb discharges; it is perhaps unnecessary to point out that the packet used should be freshly opened, and that the cotton should at all times afterwards be kept carefully protected from the dust of the room. The pads sold under the name of "antiseptic" cannot be depended on abroad, though possibly to be trusted in England where they are manufactured. The removal of a soiled pad and the application of a fresh one should be taken advantage of by the nurse for the use of the antiseptic wash. The actual washing is best done with pieces of the salicylic cotton, which must obviously never be dipped twice in the basin containing the lotion. The nurse who is interested in her cases is scarcely likely to allow any of this work, on the efficiency of which so much depends, to be done by other hands than her own. When, as usually happens, a native female, an ayah or amah, is also in attendance, this woman should on no account be allowed to take any share in these washings: she is of course ignorant of the importance of small details, and cannot be trusted to do the work either thoroughly or safely.

Ladies occasionally express a preference for native attendants to whose services they have long been accustomed, and this is only natural; but, unless in the case of native women who have undergone a special training, the doctor who knows the value of after-treatment in midwifery will always use his influence to secure the services of a European nurse for his patients. The nurse should use the ayah to assist her in changing the bed-coverings and sleeping clothes of the patient, and in such work as the serving of food and the arrangement of toilet necessaries. Native women, as a rule, handle infants skilfully, and are able to give the nurse a good deal of help in looking after the baby.

(To be continued.)

### Echoes from the Outside World.

AN OPEN LETTER TO A HOSPITAL NURSE.

OF course all of you were interested in the visit of the two Mafeking sisters to Windsor, of which mention was made in the *Nursing Mirror* last week. Not only did the Queen ask them many questions about the war, and thank them for their services, but, according to a later account, she gave a striking and pathetic illustration of the affection which exists between the Sovereign and the members of her family. One of the nurses made a simple reference to the death of Prince Christian Victor, which so touched Her Majesty that she burst into tears; and then, in words, expressed her deep grief. "His family had all been hoping that his share in the war was over and that his safe return was assured." She spoke in admiring terms of the young Prince's character, and the nurses came away much impressed with the incident.

THE latest reports as to the Tzar's illness show that the disease is taking its usual course. The fever has mostly been low, and he has been able to sleep well, which, of course, is greatly in his favour; but unfortunately typhoid patients are never really out of the wood till convalescence is far advanced. The best physicians have very judiciously been summoned from St. Petersburg, although there is at present no particular danger, except that the heart is weak. The Tzar's life is a very valuable one: he has shown himself so anxious to preserve the peace of Europe that, if anything were to happen to him, it would be a universal disaster. Our hearts go out especially just now to the Tzarina, to whose anxiety about her husband is added a fear lest her two little girls, who are at Livadia with her, should contract the same disease; and yet she dare not overtax her strength thinking of the new baby whom she hopes ere long to welcome. How devoutly she must hope it will prove to be a son! A short time back in a church dedicated to the Saint of Russian mothers, special prayers were offered for a whole week that God would bless the kingdom with an heir to the throne. The recent assault upon the German Emperor by a madwoman at Breslau has been kept from the Royal patient, lest it should unduly agitate him. The woman is said to have gone to the Law Courts to avenge herself on a lawyer who was conducting a case against her, and, failing to meet him, she threw the hatchet or chopper at the Kaiser instead. In all probability the woman will be conveyed to an asylum.

MRS. FAWCETT, who is justly regarded as the leader of the movement for the enfranchisement of women, seems to think that the prospects of the cause in the new Parliament are exceptionally good, although it has lost its champion, Mr. Faithfull Begg. She sees hope in the admission of Lord Selborne to the Cabinet, and appears to imagine that Mr. Wyndham, who is a strong supporter of the movement, will have more influence as Secretary for Ireland than he possessed as Under Secretary for War. But Mrs. Fawcett, I fancy, scarcely realises the obstacles in the way of the Government touching the question. There are at least four very solid ones, and they are severely masculine. Mr. Chamberlain, the Duke of Devonshire, Sir Michael Hicks-Beach, and Lord James of Hereford have no sympathy with the proposal to confer the suffrage on women who pay rates and taxes. Neither had Mr. Goschen, who once convulsed the House with laughter by dilating on the dire effects which would follow the admission to the franchise of "Spiers and Pond's young ladies." He has gone, it is true, but the fact that his successor is a supporter of the movement he denounced is slender ground, indeed, for the anticipation that Ministers may be induced to take it up.

THOSE of you who may have had the pleasure of seeing Chester will easily understand the suggestion of the eminent engineer, Sir Frederick Bramwell, that the new London street which is to unite Holborn to the Strand should be constructed on the lines of the beautiful Rows in that dear old city. Of course, the copy could not approach the original in beauty, because of the mellowing touch of Time; but there is much to commend in the idea. It would make the new street, architecturally, one of the best in London; it would give pedestrians a chance of shopping and promenading under cover, for the upper row of shops, standing on the top of the lower row, but much farther back, is protected, of course, by the rest of the house, which stands forward on a level with the street; and it would enhance considerably the value of the property. In Copenhagen, I believe, all the streets have their double row of shops, and there is a further advantage, that a bridge from one upper row to the other across the street would allow those who fear the danger of the street-crossing to get over in safety, and yet would be sufficiently high to allow carts to pass under. Obviously, there are difficulties in the way of steps or stone-paved slopes to reach the upper shops, not to mention others, but it would be a pity if the suggestion were discarded without the consideration it deserves, and without every effort being made to render it practicable.

DISTRICT nurses often have the power of saying a word in season about subjects which do not bear directly upon nursing. Attention has just been drawn to the late hours which the children of the present generation are allowed to keep. There used to be an idea that from seven to eight o'clock was quite late enough for children up to nine or ten years of age, but nowadays, more especially amongst the children of the poor, ten o'clock is considered quite early enough for the small people, who are running about all day and often growing fast into the bargain, to creep between the sheets. As it is pointed out, the late hours are encouraged in many cases by the clergy or the church officials who arrange eight o'clock in the evening for choir practices, although the youngest chorister is frequently not much more than six or seven years of age. This arrangement is probably the result of thoughtlessness, and could easily be altered if the evil were recognised. Meanwhile, it is impossible to lay too much stress upon the good old-fashioned proverb about "Early to bed and early to rise," and as nurses can often make suggestions which would not be tolerated from anyone else, I think the point is one which is worth thinking about.

I WONDER if you find many complaints amongst your patients respecting the want of freshness of so-called "new-laid eggs." According to the revelations lately published by the Secretary of the National Poultry Organisation Society, it would not be a matter of surprise if you did. Apart from the fact that there is always a falling-off in the supply before Christmas, although there is an increase in the demand, there is not the slightest guarantee that when a shopkeeper, whose word is to be believed, assures a purchaser that the "new-laid eggs only came in to-day," the eggs which he offers have been laid this week, or even this month. His statement may be strictly correct, but a very general custom amongst producers and higglers at this season is the holding of eggs in order to obtain better prices. From the beginning of September this is done to a much larger extent than is commonly supposed, because eggs will realise in November and December 50 per cent. more than they do in September, so long as the buyer can be deceived into accepting them as new-laid. Mr. Brown tells a story that he was once in a shop in a provincial town when a man came in, and after asking and learning what price the shopkeeper was prepared to give for a lot of eggs, he replied, "I'll keep them another week." To the knowledge of the shopkeer those eggs had been in the man's possession a month and probably longer, and might be a week or two older yet before being presented for sale across the counter. This, however, is not the worst. "Mixing" goes on to an enormous extent, country producers buying French eggs to mix amongst their "best English new-laid from our own farm." The National Poultry Organisation Society is going to organise a rapid system of collection from reliable people and stamping with a Rose-brand trade mark to show the genuineness of what they sell, in order to improve the present unsatisfactory state of affairs. But, necessarily, progress will be slow and difficult. It is to the interest of so many folks to throw cold water on a scheme which lessens ill-gotten profits.

### The Nurses' Bookshelf.

THE CENTURY INVALID COOKERY BOOK. By MARY A. BOLAND. Edited by Mrs. HUMPHREY. (T. Fisher Unwin. Price 1s.)

THIS most excellent book we have read with the greatest interest, and can thoroughly recommend it, not only to those engaged in actual nursing, but to all who have awakened to the fact "that the health and strength of a person depends largely upon what passes through his mouth; that even the turn of his thinking is modified by what he eats. This should lead all intelligent women to make food a conscientious subject of study."

Here Miss Mary A. Boland touches the keynote to three parts of the misery and unhappiness that we see around us.

As a rule, we leave our preparation of food to the most unintelligent people.

We can only hope the day is not far distant when the science of cookery, in sickness and health, and household management, will take the first place in the education of every girl that is born, letting her take up any science or art she may please after she has mastered these essentials.

Miss Boland gives us a most readable study, making the scientific side simple and easy to understand, in her "Explanatory Lessons," and follows it up by many practical and appetising recipes. We feel sure that any nurse using these will win the approbation of her patient.

The chapter devoted to "Serving" we would like to see in the hands of every woman in the kingdom, for nearly everyone comes in contact with sickness and invalids. Miss Mary A. Boland knows what is wanting in this respect when she lays such stress on the "art of serving."

Of all nurses there is no doubt that the most handicapped concerning food are those engaged in district work. Miss Mary A. Boland remembers this, and gives some valuable hints and recipes. Those having the care of quite young children are not forgotten.

The price of one shilling for this useful and instructive book is very reasonable.

LECTURES ON MEDICINE TO NURSES. By H. E. CUFF, M.D. Third edition. Pp. 280. 8vo. (London: J. and A. Churchill. 1900. Price 3s. 6d.)

A FEW months ago, in reviewing the second edition of Dr. Cuff's "Lectures," we felt compelled to point out sundry inaccuracies, ambiguities, and omissions. The early appearance of a third edition sufficiently shows that Dr. Cuff's work has met with a measure of success, but we note with pleasure that opportunity has been taken to thoroughly revise and correct the text. Although, however, no exception can now be taken to this work on the score of inaccuracy, we are still doubtful of the real wisdom of issuing what is practically a text-book of Medicine for nurses. For if a nurse wishes to read Medicine as a thing apart from Nursing, it is surely better that she should pursue her object in the pages of a treatise in which something like completeness of statement is aimed at. For, of all the sciences—or arts—there is not one so impossible of being "crammed" or "boiled down" as Medicine.

TEN LITTLE BOER BOYS. (London: Dean and Son, 160A Fleet Street).

NATURALLY, the war has influenced the Christmas literature this year, and "Ten Little Boer Boys" is quite up-to-date. It is a parody upon the well known "Ten Little Nigger Boys," and the words fit the same music as the comic song. The children, to whom the book may be given, will be pleased to recognise well-known features of the conflict, such as

"Ten little Boer boys
Firing in a line,
'Long Tom' recoiled on one,
Then there were nine."

or

"Seven little Boer boys
Full of naughty tricks,
One abused the white flag—
And then there were six."

The illustrations, which are by Mr. A. Forrest, are large in size and brilliant in colour, with plenty of life and fun, and are sure to be appreciated by the little folks. Mr. Forrest has also illustrated the time-honoured story of John Gilpin, partly in black and white and partly in colour. Here, too, the drawings are very clever, whilst the price is very low.

### Appointments.

CHILDREN'S HOSPITAL, TEMPLE STREET, DUBLIN.—Miss Gertrude Brocklehurst has been appointed lady superintendent. She was trained at Leicester General Infirmary.

CROYDON RURAL DISTRICT ISOLATION HOSPITAL.—Miss Sarah Dutton has been appointed matron. She was trained at Chester General Infirmary, and has since been matron of the Sisters' Hospital, St. Alban's.

SWANSEA GENERAL AND EYE HOSPITAL.—Miss Alice M. Barter has been appointed assistant matron. She was trained at St. Thomas's Hospital, where she has since been staff nurse, and, subsequently, sister at St. Pancras Infirmary.

PARK HILL HOSPITAL, LIVERPOOL.—Miss Edith MacCarthy has been appointed matron. She was trained at the General Infirmary, Leeds, for three years, afterwards doing two years' private nursing. Subsequently, for three and a quarter years she was sister of wards at Hull Sanatorium, and for the past five years she has been matron of the Borough Sanatorium, St. Helens.

PETERBOROUGH HOSPITAL.—Miss Edith M. Thomas has been appointed matron. She was trained at Durham County Hospital, and has since been sister in the female accident and surgical ward, Royal Infirmary, Bradford, the General Infirmary, Huddersfield, and the Beckett Hospital, Barnsley. From 1899 she has been matron at Wakefield Fever Hospital.

COUNTY HOSPITAL, YORK.—Miss Agnes Pumphrey has been appointed lady superintendent. She was trained at the General Infirmary, Leeds, and has since held the posts of sister at the Ancoat's Hospital, Manchester, and sister at The Queen's Hospital, Birmingham, matron at the Evesham Cottage Hospital, and for the last five years she has been matron of the Walsall and District, Walsall.

MARGATE COTTAGE HOSPITAL.—Miss Margaret E. Mason has been appointed nurse matron. She was trained at St. Marylebone Infirmary, and has since been sister.

### Presentations.

STRATTON COTTAGE HOSPITAL.—The lady visitors at the Stratton Cottage Hospital have presented Miss Norledge with a silver milk jug. The cup bears the following inscription:—"Presented by the lady visitors to Nurse Norledge on her leaving the Stratton Cottage Hospital after seven years' most faithful and devoted service. November 1900."

POLUISH HOSPITAL.—In consequence of the completion of the railway works at Mallaig, the hospital at Poluish, Fort William, N.B., which was opened nearly three and a-half years ago by the contractors, Messrs Robert McAlpine and Sons, has been closed, and Miss Hawkins, the matron, has been presented with a very handsome silver-fitted dressing case. The hair brushes, &c., are engraved with her monogram, and the mirror bears the following inscription:—"To Nurse Hawkins from the employés of Robert McAlpine and Sons on the Mallaig Railway. November, 1900."

### Where to Go.

TRAINED NURSES' CLUB, 11 BUCKINGHAM STREET, STRAND. Friday, November 23rd.—Lecture on "Nurse Lesions," by Miss Apell, at 7.45 P.M.

NATIONAL ORTHOPŒDIC HOSPITAL, 234 GREAT PORTLAND STREET. Friday, November 30th.—Opening of the annual sale of work by the Duchess of Marlborough, at 2 P.M.

HAMPSTEAD CONSERVATOIRE, SWISS COTTAGE. Monday December 17th.—Reading of Hamlet by Mr. Forbes-Robertson, at 8.30 P.M., in aid of the Hampstead Hospital.

### Wants and Workers.

A NURSE has a portion of uniform (including a last year's blue winter cloak, large size) for which she has no further use, and she will be glad to give it to anyone needing the things. Particulars from "Nurse," 74 Fairhazel Gardens, Hampstead, N.W.

### Everybody's Opinion.

### THE SPARTAN NURSE IN PRIVATE WORK.

"ONE WHO LOOKS ON" writes: I am amused to find that someone who signs herself "One Who Looks on Also" has fitted the case I mentioned to an individual she knows personally! But I am afraid the cases do not fit. In the case *I* wrote of the nurse tried no expedients to relieve the patient's backache; declined entirely to wash the patient's face (not at 7 p.m., but at 9 p.m.), and there was no question of "a quickly-devoured dinner" to be digested, as the patient was on milk diet. I have been a nurse myself, and I still plead for less Spartan severity, and more careful observation of the individual needs of patients. Gentleness as well as firmness is an essential part of good nursing.

### THE CRISIS AT CROYDON.

"AN OLD CROYDON NURSE" writes: I was very glad to see the step Miss Julian took in refusing to sign the certificates of training of the three nurses who had trained under her at the Croydon Infirmary. As an old Croydon nurse I can testify to the great good Miss Julian has done, and the most excellent nurses she has turned out during the number of years she has been at the Croydon Infirmary. It would be a gross injustice to those nurses if certificates were given broadcast to nurses who did not deserve them. I contend that the matron is the best judge as to whether nurses under her charge are deserving of certificates or not. I take it that a mistress applying for a servant's character would not appeal to the master of the house. The Guardians do not realise the injustice they are doing to the present nurses training in their infirmary, as no post is worth accepting where the matron's reference or signature is not required, especially one so popular in the nursing world as Miss Julian.

### NURSE DOWLING'S ASSERTIONS.

"A CONTENTED NURSE" writes: Surely Miss Dowling has been particularly unfortunate to have worked in six institutions where the treatment of nurses is so uniformly bad. After five years in two large London hospitals, I have come to the conclusion that the matron is usually harder worked than any other woman in the institution, but have always found her ready to give help or a word of encouragement to any of her "bullied and slave-driven" probationers, even in regard to private affairs in no way connected with hospital life. As to "nurses doing the work of three women" while "matrons sit in easy chairs," intending probationers may like to know of an incident in my own experience. While a probationer, in my first year, I took scarlet fever. Owing to influenza being prevalent nearly every ward was short of nurses, and to prevent more work being thrown on them, the matron herself did everything for me till the following afternoon, when an "outside" nurse arrived. I believe my experience is not at all exceptional, and if there is anything hard or unfeeling in the treatment of nurses, on inquiry the board of management, and not the matron or medical officers, will generally be found responsible.

"A LIVERPOOL INFIRMARY-TRAINED NURSE" writes: Nurse Dowling pleads on behalf of her fellow-workers and nurses generally, but I for one would be exceedingly sorry to think that any woman worthy of the name, would echo her most un-nurselike sentiments. She states that "we are all at the mercy of those who employ us," but nurses who work conscientiously need not fear those in authority. Treating all with the respect due to them, they will accept their superior judgment gained by long experience in hospital matters. Matrons are accused of sitting in easy chairs and reading novels. Surely, this is no crime, and any nurse may do likewise if she so chooses in her off-duty time. Indeed, a good healthy novel may tend to broaden our views and possibly make us more charitable in our remarks about our fellow creatures. I can speak of a pro's. life as one trained in a large provincial infirmary. On duty there is any amount of real hard work, but off duty the life is as happy as each individual pleases to make it. The food is plain and wholesome, and there is plenty of it. If any of the nurses are ill, they are treated with the greatest kindness, and no member of the staff is expected to go on duty if she does not feel fit for work. I am surprised that Nurse Dowling should stay two years in a home to be barbarously treated, and she ought to have inquired about testimonial before joining it. I trust that intending probationers will not allow a bitter letter written by one who has so many grievances to alter their plans. My advice to them is Go in for nursing, join a hospital or infirmary, being a recognised training school, be loyal to your matron, doctors, and school.

"NURSE NORAH" writes: May I have space for a few lines in reference to Miss Agnes Dowling's letter? I feel naturally sorry for Nurse Dowling; her letter is so full of sadness and of bitterness. I have no intention of criticising either Dr. Dod's statement or the Judges' decision. They are the powers that be, and perhaps were I in Nurse Dowling's place, I too might feel her bitterness; but, I hope, not to the extent of making such sweeping assertions as to the ills the nursing flesh is heir to. In all lines of life there are times when we must bend to the storms passing over us. We have strong contrary winds to contend with; but I think that, taking the nursing profession as a whole, once fairly launched and over the breakers of probation it is pretty fair sailing. I have had a good deal of private nursing, and now have nothing to depend upon but what my work brings me; but I have never considered myself underpaid, underfed, or slave-driven—indeed, I have been very generally well treated. I think we carry much of our own happiness and comfort about with us in bowing to circumstances. The discipline of probationary days is as valuable as gold. A nurse's first duty, like the soldier's, is to obey, whether doctor, matron, or sister; afterwards the world and patients accept one much at one's own value. So I advise probationers to go in and win a good nurse's name, than which there is no better. Whether in making more bearable a sick-bed wearisome if not serious; striving to draw back hesitating steps from the edge of the grave, or soothing the last hours of the dying, the nurse's life is a grand one, well worth the minor difficulties, be they many or few. Nursing, like virtue, is its own reward.

### THE PAY OF IRISH NURSES.

"SISTER CATHERINE" writes: Your note interested me greatly, as I for many years nursed in Ireland and thought with some of my Irish sisters that the pay was too small—forgetting, or not appreciating, the many little things besides salary that go to make one content. I belonged to an institution which was home, where the matron and many of the nurses gave you the flattering impression that they were personally interested in you. If you were ill you had every care, and I have known cases of nurses being sent to the seaside to regain strength without being made to feel under any obligation. Not the least consideration was that when we were in the home we had good wholesome food, and not being afraid to breathe or speak during meal time, we were natural and happy. My salary was £25 a year paid quarterly, out of which I had to buy bonnet and cloak. Well, I set off to England to make my fortune and felt that I had endless riches with £35 a year and full uniform. But the food was wretched. Most of the nurses were in the habit of buying food and eating it in their bedrooms. At first I felt very much ashamed, but when one feels run down for want of it there is an excuse. This makes a big hole in the pay, and though my experience may be an exception, it may be worth while for others to think of their present advantages before yielding to the tempting bait of high salary.

### THE MADRAS GENERAL HOSPITAL.

"PRO BONO PUBLICO" writes: Your correspondent "Another Who Knows the Hospital" cannot, judging from the statement that the nursing department of the Madras General Hospital is worked on an "ideal basis"! know much of any other hospital. Some weeks ago "One Who Suffered" pointed out the difficulty and expense incurred by a nurse who wished to appeal against the arbitrary decision of her immediate superior, and while such a state of things exists the hospital can hardly be considered as worked on an "ideal basis." "Another Who Knows the Hospital" says "the nursing staff, with the exception of the matron and assistant matron, who receive very good salaries, are entirely *Eurasian, Anglo-Indian, or native.*" This is very incoherent, as the whole population of India is, I suppose, composed of the three classes mentioned, and it would be interesting to know of what nationality are the matron and assistant matron, or what connection their "good salaries" have with the rest of the sentence quoted. Then "Another Who Knows the Hospital" complains that English nurses expect too much in the way of "pay, amusements, lack of all restriction, and a minimum of work." I think I have known most of the English ladies who at different times have worked in the Madras General Hospital, and they have all complained of the lack of definite rules on the subject of amusements and leave out of hospital; for, while some could obtain as much as they wanted, others, less favoured, could get none at all. Then as to English nurses wanting a "minimum of work," this statement is hardly reconcilable with the fact that the English nurses have always been promoted over the heads of Eurasians on the ground that they are more conscientious and do *better* work. No one in Madras for a moment supposes that Government is unwilling to do much for the nursing staff of the General Hospital, and it is for this reason that all who know the exact state of affairs in that institution are so anxious that Government should institute an impartial and searching inquiry which would disclose all that now keeps nurses of *the right sort* from joining the Hospital. It is gratifying to learn that, even if it does no further good, the way in which the Madras General Hospital has been brought to public notice through the Hospital Nursing Mirror has had the immediate effect of improving the condition of the nurses in many little ways, and it is sincerely hoped that these may only be forerunners of permanent improvements on a large scale.

### The Metropolitan Nursing Association.

### THE ADDITIONS TO THE HOME AT BLOOMSBURY.

By A Correspondent.

"No, there is no speechifying," Mrs. Minett, who received the visitors on behalf of the committee of the Metropolitan Nursing Association on the occasion of the reopening of the Home at Bloomsbury, said in answer to my inquiry: "You see we have no rooms large enough at Bloomsbury, and, besides, everybody knows all there is to be said about the work of the Association; they hear it at the annual meetings. We think it very much better to have an informal gathering like this."

The guests assembled on Tuesday in the pretty sitting-room, decorated some years ago by the Kyrle Society, in blue-green paint with a deep frieze of natural flowers. The entire house has been freshly painted and papered to be in keeping with the new rooms, and pale blue greens predominate, relieved by blue and white curtains of washable material. The colouring is both harmonious and restful to the eye—a great consideration in a nursing home, and indeed, in any place to which tired people return after a hard day's work. The additions consist of four bedrooms, with bathrooms and lavatories; the dining room, in which lectures are given, has been made lighter by means of an extra skylight; and a very uninhabitable bathroom downstairs has been converted into a most convenient district room, with drying cupboard for wet clothes, sinks with specially grooved boards for scrubbing mackintoshes, a rack for strong boots, and various other arrangements for lightening labour when the nurse comes in from her district.

### A Plague of Rats.

"We were infested with rats," Mrs. Minett told me; "but we hope we have got rid of them now." And, indeed, everything looked so bright and clean and wholesome that I thought he would be a bold rat that ventured to show a whisker in No. 23; and his reception would probably not be encouraging to further efforts. Another improvement is the installation of electric light throughout the house; and in one of the bedrooms I noticed a hot-air radiator, while another has a gas-heating stove.

### The Guests.

A large number of guests were present, and one of the superintendents was heard congratulating herself on it being a fine day, which it was, all things considered, for the rain kept off pretty well for November. Among those present were Miss Gordon, the Matron of St. Thomas's Hospital; and with her was Miss Haig Brown, of the Nightingale Home; Miss Peter, Miss Percy (Superintendent of the Paddington District Nursing Home), and, indeed, many of the district superintendents; Mrs. W. C. Cane, Miss Louisa Twining, Miss Rosalind Paget, Miss MacVickers, Miss Dods, and others. Many gentlemen were also present, among them the Rev. Dacre Craven, representing the committee; Mr. Harold Boulton, Treasurer of the Queen's Commemoration Fund; Mr. Bonham Carter, the Rev. Mr. Russell (Warden of St. Bartholomew's Guild), the Rev. Sir Borradaile Savory (Rector of St. Bartholomew-the-Great, Smithfield), the Master of St. Katherine's, many local clergy, and others. Some good instrumental music was given.

### Middle-class Nursing.

From Miss Dods I heard incidentally something about the experiment which is being tried by herself and Miss Thorn (formerly a Bloomsbury nurse) in nursing among the middle classes. They have only been working a few months; but they are quite convinced that there is a real need for nursing among, for example, ladies living in flats or lodgings, earning their living, and perhaps not ill enough to go into a hospital, and certainly not well off enough to pay the fees of an ordinary private nurse. The difficulty is in finding the right cases.

"It is a most useful work," said Mrs. Minett, "and our Superintendent here (Miss Haddon), loses no time in sending suitable cases to John Street Vicarage, where the two ladies live; for there are many who have no right to the district nurse, and who yet cannot pay high fees. We wish the work every success."

The nurses, of whom there are at present ten attached to the Association, are very glad to be able to get back to their own quarters. During the five months or so that the alterations have being going on, they have been living in Russell Square.

### To Nurses.

We invite contributions from any of our readers, and shall be glad to pay for "Notes on News from the Nursing World," or for articles describing nursing experiences, or dealing with any nursing question from an original point of view. The minimum payment for contributions is 5s., but we welcome interesting contributions of a column, or a page, in length. It may be added that notices of entertainments, presentations, and deaths are not paid for, but, of course, we are always glad to receive them. All rejected manuscripts are returned in due course, and all payments for manuscripts used are made as early as possible at the beginning of each quarter.

### TRAVEL NOTES AND QUERIES.

Pontresina and St. Moritz (M. C. N.).—These places are close together, and neither has a winter season; from the middle of June to middle of September is the usual time for visiting them. Pontresina takes no winter visitors, nor does St. Moritz, but 200 feet above St. Moritz lies St. Moritz Dorf, which, from its altitude, escapes the dampness and sharp winds of St. Moritz itself. At the Dorf there is a winter season, which is beginning to compete with that of Davos. The climate of Pontresina and St. Moritz do not differ much, but you must consult your medical man. Pontresina does not encourage invalids, and is in no way a winter station.

### For Reading to the Sick.

"In the morning, then, ye shall see the glory of the Lord."—*Ex.* xvi. 7.

Oh, timely happy, timely wise,
Hearts that with rising morn arise !
Eyes that the beam celestial view
Which evermore makes all things new !

Old friends, old scenes, will lovelier be
As more of heaven in each we see :
Some softening gleam of love and prayer
Shall dawn on every cross and care.

As for some dear familiar strain
Untired we ask, and ask again,
Ever in its melodious store
Finding a spell unheard before.

Such is the bliss of souls serene,
When they have sworn, and steadfast mean,
Counting the cost, in all to' espy
Their God, in all themselves deny.

Oh, could we learn that sacrifice,
What lights would all around us rise !
How would our hearts with wisdom talk
Along life's dullest dreariest walk !—*Keble.*

Lord, I my vows to Thee renew ;
Disperse my sins as morning dew ;
Guard my first springs of thought and will,
And with Thyself my spirit fill.—*Thomas Ken.*

Morning by morning think, for a few moments, of the chief employments of the day, any one thing of greater moment than others, thine own especial trial, any occasions of it which are likely to come that day, and by one short strong act commend thyself beforehand in all to God ; offer all thy thoughts, words, and deeds to Him—to be governed, guided, accepted by Him. . . Choose some great occasions of the day, such as bring with them most trial to thee, on which, above others, to commend thyself to God.—*E. B. Pusey.*

The heights of Christian perfection can only be reached by faithfully each moment following the Guide who is to lead you there, and He reveals your way to you one step at a time, in the little things of your daily lives, asking only on your part that you yield yourselves up to His guidance. If, then, in anything you feel doubtful or troubled, be sure that it is the voice of your Lord, and surrender it at once to His bidding, rejoicing with a great joy that He has begun thus to lead and guide you.—*H. W. S.*

It matters not where or what we are, so we be His servants. They are happy who have a wide field and great strength to fulfil His missions of compassion ; and they, too, are blessed who, in sheltered homes and narrow ways of duty, wait upon Him in lowly services of love. Wise or simple, gifted or slender in knowledge, in the world's gaze or in hidden paths, high or low, encompassed by affections and joys of home, or lowly and content in God alone, what matters, so that they bear the seal of the living God ? Blessed company, unknown to each other, unknowing even themselves !—*H. E. Manning.*

Will you not, before venturing away from your early quiet hour, "commit thy works" to Him definitely, the special things you have to do to-day, and the unforeseen work which He may add in the course of it ?—*F. R. Havergal.*

Build a little fence of trust
Around To-day :
Fill the space with loving work
And therein stay ;
Look not through the sheltering bars
Upon To-morrow.
God will help thee bear what comes
Of joy or sorrow.—*M. F. Butts.*

### Notes and Queries.

**The Editor is always willing to answer in this column, without any fee, all reasonable questions, as soon as possible.**

**But the following rules must be carefully observed :—**

1. **Every communication must be accompanied by the name and address of the writer.**
2. **The question must always bear upon nursing, directly or indirectly.**

**If an answer is required by letter a fee of half-a-crown must be enclosed with the note containing the inquiry.**

*Nursing Abroad.*

(79) Can you tell me the usual fees charged by nurses (three-year certificate and midwife) abroad ?—*Nurse.*

From 10 frs. per day ; maternity, 300 frs. per month.

*Address Wanted.*

(80) Is there a directory published containing the names and addresses of male nurses ? I wa t to trace a man named Edgar or his wife ; both were trained nurses.—*Mater.*

There is no such directory.

*Advertising.*

(81) Having had fourteen months' experience as matron and superintendent of an institution fo the open-air treatment of consumption I should be glad if you could tell me in which medical publications it would be best to advertise for a similar post. I am a certificated nurse, and hold the L.O.S. certificate.—*Adda.*

The Mirror has an excellent reputation as an advertising medium for all kinds of nursing appointments.

*Abroad.*

(82) I am anxious to go abroad, and should be glad to know if there are openings for nurses in Australia or Canada. Would it be best to train in England or in one of these places ? (2) Is there any hospital which trains for L.O.S. without payment ?—*Mavourneen.*

The places are so different as to be hardly comparable. It is decidedly advantageous to be trained amongst the people with whom you intend to live. The Royal Victoria Hospital, Montreal, Quebec, and the Winnipeg General Hospital Training School for Nurses offer favourable terms. Write to the Lady Superintendents for particulars. (2) No.

*Nursing Association.*

(83) Could you oblige me with the address of the association of certificated nurses in West London to which allusion was made in the Mirror of July 21st ? I live in a flat, and such an association would be an immense boon to myself and my neighbours. I am also making enquiries into the question of cheaper nursing homes for the middle classes, and would be grateful for any information on the subject.—*A. Z.*

The association alluded to was the West London Association of Certificated Nurses, Hammersmith Road, W. Many associations now add a visiting nurse to their staffs, whilst the Nurses' Hostel, Francis Street, N.W., has pursued this system for some time. Private nursing homes are difficult themes on which to generalise. Each has its own merits or defects from the point of view of the individual patients using it.

*Work for the Poor.*

(84) Could you tell me where I could be well trained as nurse for work amongst the poor ? Are there any hospitals where nurses are allowed to sit down sometimes, as I am not strong enough for an ordinary London hospital ? I should be glad of some advice.—*Worker.*

Your best plan would be to train at a small hospital. Beware, however, of bogus institutions. See advertisements, or advertise in the Mirror. Nurses are more considerately treated than you imagine.

*Antiseptics.*

(85) I should be glad to know if, having supplied all antiseptics, medicines, dressings, &c., I am entitled to charge for them when sending in my account at the end of engagement ?—*H. R. A.*

Certainly you are entitled to receive back all outlay on your patient's behalf. We should, however, recommend you strongly not to supply medical appliances. Grave abuses may creep into such a practice, and it is liable to cause unpleasantness between employer and employed.

*Qualification.*

(86) Will you kindly tell me if three years' training in a union hospital will qualify me to become a Queen's nurse ? Is the medical and surgical training efficient, and are the operations good ?—*Nurse Eva.*

Yes. The training in many such institutions ranks very highly.

*Private Operation.*

(87) Would anyone inform me what a doctor will require for an operation on hæmorroids ; and also how to prepare a T bandage for the same ?—*Grateful.*

See Notes on "Rectal Nursing" recently published in the Mirror.

*Sanitary Inspector.*

(88) Is there any institute in Manchester where I could qualify as Sanitary Inspector ? I am already a fully-qualified nurse.—*Nurse W.*

You could obtain particulars from the Manchester Ladies' Health Society. Write to Mrs. Hardie, the Cliffe, Broughton.

**Standard Books of Reference.**

"The Nursing Profession : How and Where to Train." 2s. net ; post free, 2s. 4d.
"The Nurses' Dictionary of Medical Terms." 2s.
"Burdett's Series of Nursing Text-Books." 1s. each.
"A Handbook for Nurses." (Illustrated.) 5s.
"Nursing : Its Theory and Practice." New Edition. 3s. 6d.
"Helps in Sickness and to Health." Fifteenth Thousand.
"The Physiological Feeding of Infants." 1s.
"The Physiological Nursery Chart." 1s. ; post free, 1s. 3d.

All these are published by The Scientific Press, Ltd., and may be obtained through any bookseller or direct from the publishers, 28 and 29 Southampton Street, London, W.C.

